# Impact of dalteparin sodium and luteal phase support on serum markers, interleukins, and pregnancy outcomes in patients with in vitro fertilisation failure

**DOI:** 10.5937/jomb0-57360

**Published:** 2026-01-28

**Authors:** Jinmei Lei, Ye Jin, Nv Geng, Huijuan Zhang

**Affiliations:** 1 Tangshan Maternal and Child Health Care Hospital, Department of Reproduction, Tangshan, Hebei Province, China

**Keywords:** in vitro fertilization-embryo transfer, assisted reproduction, clinical pregnancy rate, dalteparin sodium, luteal phase support, serum markers, interleukins, CA-125, IL-6, in vitro oplodnja-transfer embriona, asistirana reprodukcija, stopa kliničke trudnoće, natrijum-dalteparin, podrška u lutealnoj fazi, serumski markeri, interleukini, CA-125, IL-6

## Abstract

**Background:**

In vitro fertilization-embryo transfer (IVF-ET) remains a challenging treatment for infertility, particularly in patients with repeated implantation failures. Dalteparin sodium (DS), a low-molecular-weight heparin, has shown promise in improving IVF outcomes due to its antithrombotic and immunomodulatory properties. This study evaluated the impact of DS combined with luteal phase support (LPS) on serum markers, interleukins, pregnancy outcomes, quality of life (QOL), and social support in women with prior in vitro fertilisation (IVF) failures.

**Methods:**

This retrospective study included 80 patients with a history of repeated IVF failure who underwent assisted reproduction at our centre. Patients were randomised to receive either LPS alone (Ctrl; n = 40) or LPS plus daily subcutaneous DS (Exp; n=40). Serum levels of b-hCG, FSH, E2, LH, CA-125, and interleukin-6 (IL-6) were measured and compared between the groups.

**Results:**

The Exp group demonstrated significantly higher clinical pregnancy rates (CPR) and embryo implantation rates (EIR) compared to the Ctrl group (P &lt; 0 .0 5 ). Additionally, the miscarriage rate (MCR) was significantly lower in the Experimental group (P&lt; 0.05). Moreover, the Exp group exhibited significantly lower serum levels of CA-125 and IL-6 compared to the Ctrl group (P&lt; 0.05). Improvements in quality of life (QOL) measures were also observed in the Exp group, with significant increases in physiological function, social function, emotional function, and mental health (P&lt; 0.05). No significant difference in the incidence of adverse reactions (ARs) was found between the two groups (P&gt; 0.05).

**Conclusions:**

The combination of LPS and DS appears to be a safe and effective strategy for improving pregnancy outcomes and QOL in patients with repeated IVF failure. This combined treatment may exert its beneficial effects by modulating key serum markers and interleukins, such as CA-125 and IL-6, involved in implantation and pregnancy maintenance.

## Abstract (sr)

**Uvod:**

In vitro oplodnja sa transferom embriona (IVF-ET) je i dalje izazovan tretman za neplodnost, naročito kod pacijentkinja sa ponovljenim neuspesnim pokusajima implanta-cije. Natrijum-dalteparin (ND), heparin niske molekulske mase, je pokazao obećavajuće rezultate u poboljsanju isho-da IVF-a zahvaljujući svojim antitrombotičnim i imuno-modulatornim svojstvima. Ova studija je ispitivala efekte ND u kombinaciji sa podrskom u lutealnoj fazi (LPS) na serumske markere, interleukine, ishode trudnoće, kvalitet života (QOL) i socijalnu podršku kod žena sa prethodnim neuspesima u IVF.

**Metode:**

U ovoj retrospektivnoj studiji je učestvovalo 80 pacijentkinja sa istorijom ponovljenog neuspeha kod IVF-a koje su se podvrgle asistiranoj reprodukciji u našem centru. Pacijentkinje su nasumično podeljene u dve grupe: jedna je primala samo LPS (kontrolna grupa; n=40), a druga je primala LPS uz dnevnu potkožnu primenu ND (eksperimentalna grupa; n=40). Mereni su i upoređeni serumski nivoi b-hCG, FSH, E2, LH, CA-125 i interleukina-6 (IL-6).

**Rezultati:**

Eksperimentalna grupa je pokazala značajno višu stopu kliničke trudnoće (CPR) i stopu implantacije embriona (EIR) u poređenju sa kontrolnom grupom (P&lt; 0,05). Takođe, stopa spontanih pobačaja (MCR) bila je značajno niža u eksperimentalnoj grupi (P&lt; 0,05). Pored toga, serumski nivoi CA-125 i IL-6 bili su značajno niži u eksperimentalnoj grupi u poređenju sa kontrolnom (P&lt; 0,05). Primećena su i poboljšanja u merama kvaliteta života (QOL) u eksperimentalnoj grupi, uključujući značajna poboljšanja fiziološke funkcije, fiziološke uloge, socijalne funkcije, emocionalne funkcije i mentalnog zdravlja (P&lt; 0,05). Nije bilo značajnih razlika u učestalosti neželjenih reakcija (AR) između dve grupe (P&gt; 0,05).

**Zaključak:**

Kombinacija LPS i ND se pokazala kao bezbedna i efikasna strategija za poboljšanje ishoda trudnoće i kvaliteta života kod pacijentkinja sa ponovljenim neuspesima kod IVF-a. Ovaj kombinovani tretman može da pruži svoje korisne efekte modulacijom ključnih serumskih markera i interleukina, kao što su CA-125 i IL-6, koji su uključeni u implantaciju i održavanje trudnoće.

## Introduction

In vitro fertilisation (IVF) with embryo transfer (ET) - sometimes referred to historically as »test-tube baby« technology - [1] is a procedure in which oocytes and sperm are collected from subfertile couples and fertilised ex vivo. After the embryos develop, they are transferred into the uterine cavity to achieve pregnancy [2] [3]. Although fertilisation occurs ex vivo, IVF replicates key physiological steps of natural conception, allowing for early embryo development before being transferred to the mother's uterus [4]. Compared to natural conception, IVF success rates are substantially higher. With the advancement of biomedical technologies, improvements in culture media, and the expertise of medical professionals recently, the success rates of IVF worldwide have increased from around 20%-25% to 60% or even higher [5] [6]. According to medical experts, among natural conception, artificial insemination, and TTB, the success rate of TTB is the highest. This technique is primarily suitable for cases of female infertility, such as tubal adhesions, blockages, endometriosis, abnormal follicle development, and ovulation disorders [7]. Despite these gains, a subset of patients still experience repeated implantation failure (RIF). Recent studies have therefore investigated immunological and biochemical biomarkers, such as CA-125 and IL-6, as potential predictors of endometrial receptivity and IVF success.

Dalteparin sodium (DS) is a low molecular weight heparin (LMWH) that exhibits anticoagulant and thromboprotective effects by inhibiting thrombin and clotting factors. Clinically, DS is primarily used for preventing and treating disorders related to thrombosis. It can be utilised to prevent or treat deep vein thrombosis and is recommended for high-risk patients undergoing surgical or orthopaedic procedures [8] [9]. Additionally, DS can be employed for anticoagulation during extracorporeal circulation in patients with renal insufficiency and for anticoagulant therapy in cardiovascular diseases, such as myocardial infarction [10]. In addition to its anticoagulant effects, DS has been shown to modulate the levels of specific serum markers and interleukins, which may contribute to its beneficial effects on in vitro fertilisation (IVF) outcomes. When using DS, the crucial point is to monitor the patient for signs of bleeding closely, and patients should be vigilant in reporting any bleeding symptoms to their healthcare provider [11] [12]. The corpus luteum is a cellular cluster formed from the follicular wall and granulosa cells after ovulation. It has a secretory function and appears yellow. The corpus luteum produces hormones that stimulate the secretion of endometrial glands, promote spiral artery development, maintain endometrial thickness, and facilitate embryo implantation [13] [14]. Insufficient corpus luteum function can impact the secretion of estrogen and progesterone in females. This can result in inadequate endometrial transformation, leading to decreased chances of embryo implantation and increased risk of miscarriage [15]. Moreover, the levels of specific serum markers and interleukins, such as CA-125 and IL-6, have been associated with corpus luteum function and endometrial receptivity. Therefore, the treatment of corpus luteum function in patients can significantly affect the success rates of IVF and embryo transfer (ET).

In conclusion, IVF and ET techniques have provided hope for many infertile couples, with continuously improving clinical success rates. However, there are still some patients who experience embryo implantation failure due to various factors affecting the mother and the embryo. Therefore, data were collected from 80 patients who experienced repeated IVF failure and requested assisted reproduction on October 5, 2019, and December 1, 2022, at the Reproductive Medicine Center of the hospital. These patients were grouped into an experimental (Exp) group and a Control (Ctrl) group, with n 40. The Ctrl group received luteal phase support (LPS), while the Exp group received LPS + DS. The objective of this work was to analyse in depth the intervention effect of LPS + DS on assisted reproduction in patients with repeated IVF-ET failure. Furthermore, this study aimed to investigate the impact of LPS + DS on the levels of key serum markers and interleukins, including CA-125 and IL-6, to elucidate their potential role in improving IVF outcomes.

## Materials and methods

### Research objects

Eighty patients who experienced repeated IVF failures and requested assisted reproduction were retrospectively collected from the Reproductive Medicine Center at Tangshan Maternal and Child Care Hospital from October 5, 2019, to December 1, 2022. They were grouped into an experimental (Exp) group and a control (Ctrl) group, with 40 patients in each. The assignment to the groups was randomly performed based on different treatment methods.

### Inclusion criteria

The study participants provided informed consent after obtaining consent from their family members. The ethics committee has approved the implementation of this study.

The patients enrolled had to satisfy all the following conditions: I. patients who had undergone IVF-ET and experienced repeated IVF failure with more than 3 embryo implantation attempts; II. patients who had a total of 10 high-quality embryos transferred without achieving implantation; III. patients aged below 38 years; IV. patients with complete baseline data, and V patients who voluntarily opt for assisted reproduction again.

### Exclusion criteria

The patients had to be excluded from this work if they had any of the below conditions: I. patients with chromosomal abnormalities; II. patients with hydrosalpinx (blockage or dilation of the fallopian tubes); III. patients with underlying conditions such as hypertension, diabetes, or other chronic diseases; IV patients with bleeding disorders, and V. those with hearing or visual impairments.

### Treatment methods

In the Ctrl group, patients were administered LPS and instructed to take four tablets of oral fen-materol red tablets per day, starting from the third day of their menstrual cycle. After one week of medication, patients underwent a B-ultrasound examination (BUE). If the thickness of the endometrium exceeded 8 mm, patients continued taking four tablets of fenmaterol red tablets per day. Additionally, patients were given 85 mg of progesterone vaginal sustained-release gel daily. The use of progesterone allowed for ET on the sixth day.

In the Exp group, patients received LPS and, starting from the day of ET, were administered a subcutaneous injection of 5000 IU of DS once daily. On the 35^th^ day after ET, a BUE was performed to observe the embryo and fetal heart. At this point, the administration of DS can be discontinued. During the medication period, patients underwent weekly monitoring of platelet count, coagulation function, liver function, and kidney function, and any abnormalities were promptly addressed.

Rey and Rivard (2000) showed that daily 5,000 IU dalteparin maintains stable anti-Xa levels throughout pregnancy without third-trimester dose adjustments. Although specific IVF data are limited, these findings support the use of 5,000 IU/day in IVF patients [16].

### Observation indicators

General information about all patients was collected, including age, body mass index (BMI), number of previous miscarriages, number of transferred embryos, and educational level (primary school or below, high school or vocational school, or college or above).

Two weeks after ET, fasting venous blood samples were collected from patients to measure levels of beta-human chorionic gonadotropin (β-hCG), follicle-stimulating hormone (FSH), estradiol (E2), luteinising hormone (LH), and other relevant hormones. To further investigate the immunological factors involved in implantation and pregnancy, serum levels of CA-125 and interleukin-6 (IL-6) were also measured. CA-125 was quantified using chemiluminescent microparticle immunoassay (CMIA) using the ARCHITECT Ca 125 II assay, while IL-6 levels were determined using high-sensitivity ELISA.

Furthermore, five weeks after ET, patients would undergo a BUE to observe the presence and number of gestational sacs in the uterine cavity. The clinical pregnancy rate (CPR), embryo implantation rate (EIR), and miscarriage rate (MCR) were calculated.


(1) CPR=\frac{Number \ of \ pregnancy \ cycles}{Number \ of \ transplant \ cycles} \times 100 \%

(2) EIR=\frac{Number \ of \ implanted \ embryos}{Number \ of \ transplanted \ embryos} \times 100 \%

(3) MCR=\frac{Number \ of \ abortion \ cycles}{Number \ of \ pregnancy \ cycles} \times 100 \%

Next, the quality of life (QOL) of patients in both groups was assessed before and after treatment using the MOS Short Form Health Survey (SF-36). This survey comprised 36 questions and addressed multiple areas, including physical functioning, role restrictions due to physical health, bodily pain, overall health perception, vitality, social functioning, role limitations due to emotional issues, and mental health (MH).

The level of social support in both groups was assessed using the Social Support Rating Scale (SSRS). The participants included were adults aged 16 and above, individuals with recovered severe mental illness, individuals with neurotic disorders, and individuals with physical illnesses. The scale consisted of 10 items, including 3 items (2, 6, and 7) relevant to objective support, 4 items (1, 3, 4, and 5) relevant to subjective support, and 3 items (8, 9, and 10) relevant to social support, respectively.

Eventually, the level of adaptability in both groups will be assessed using the Chinese version of the Family Adaptability and Cohesion Evaluation Scale 2^nd^ Edition (FACESIICV). This self-report scale evaluated both cohesion (emotional bonding among family members) and adaptability (the family system's ability to change according to specific family circumstances and different stages).

### Methods for statistics

Data were analysed using SPSS 19.0. Descriptive statistics were utilised to represent continuous variables as mean±standard deviation (mean±SD), while the categorical variables were presented as percentages (%). Repeated measures analysis of variance (ANOVA) was performed for between-group comparisons and two-way ANOVA for within-group comparisons. P<0.05 was used for two-tailed tests to determine statistical significance.

## Results

### Baseline characteristics

**Figure 1:**
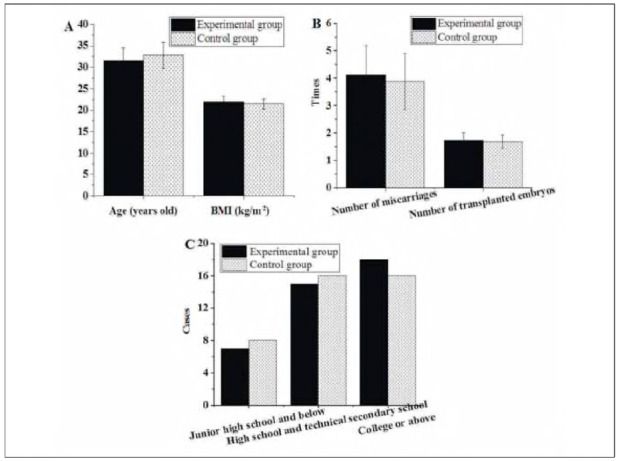
Basic introduction of patients in different groups.<br>(A: age and BMI; B: history of miscarriages and ET; C: education level)

As illustrated in Figure 1, In the experimental (Exp) group (n = 40), the mean age was 31.55±2.95 years, the mean BMI was 21.92 ±1.33 kg/m^2^, the mean number of prior miscarriages was 4.12±1.06, and the mean number of previous ET was 1.73±0.28. In terms of education level, 7 patients had completed junior high school or below, 15 had completed high school or vocational school, and 18 had completed college or higher education. On the other hand, the patients in the Ctrl group were 32.74±3.06 years old on average, had a BMI of 21.45±1.18 kg/m^2^, miscarriages of 3.88±1.02 times, and an average number of ET of 1.69±0.24. In terms of education level, 8 patients had completed junior high school or below, 16 had completed high school or vocational school, and 16 had completed college or higher education. There were no significant differences between groups in terms of age, BMI, miscarriage history, number of ETs, or educational level (all P>0.05).

### Pregnancy outcomes of patients in different groups

**Figure 2:**
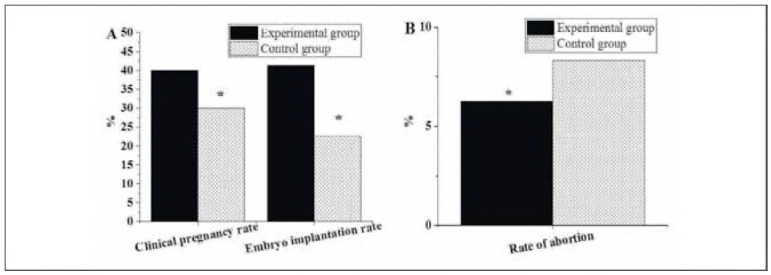
Pregnancy outcomes of patients in various groups (A: CPR and EIR; B: MCR).<br>Note: * suggested a great difference with P < 0.05 in the comparison

As given in Figure 2, the CPR in the Exp group was 40%, the EIR was 41.25%, and the MCR was 6.25%. In the Ctrl group, the CPR, EIR, and MCR were 30%, 22.62%, and 8.33%, respectively. It was evident that the CPR and EIR of the Exp group were significantly higher, while the MCR was lower, indicating notable differences compared to those in the Ctrl group (P<0.05).

### Comparison of post-treatment hormone levels of patients

**Figure 3:**
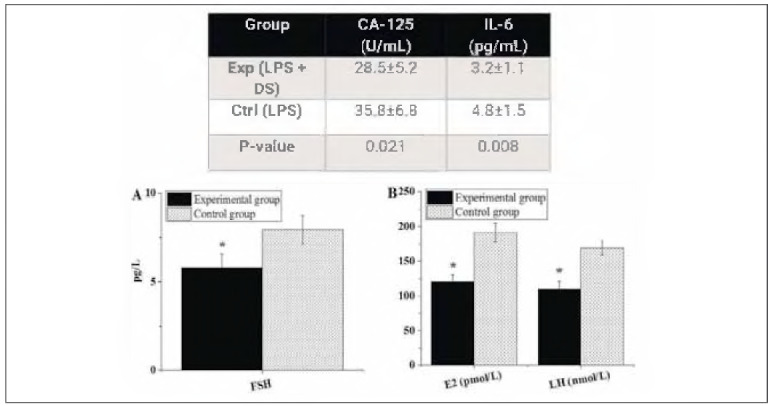
Post-treatment hormone levels of patients (A: FSH; B: E2 and LH).<br>Note: * suggested a great difference with P < 0.05

Figure 3 below compares the post-treatment hormone levels of patients after different treatments. The FSH level in the Exp group was 5.81±0.76 pg/L, the E2 level was 120.75±9.81 pmol/L, and the LH level was 109.48±11.52 nmol/L. In the Ctrl group, the FSH, E2, and LH levels were 7.93±0.82 pg/L, 191.14±13.46 pmol/L, and 169.22±10.05 nmol/L, respectively. It was evident that the FSH, E2, and LH levels of patients receiving LPS + DS were significantly lower than those treated with LPS alone, presenting notable differences (P<0.05).

### Serum levels of CA-125 and IL-6

Table 1 presents the serum levels of CA-125 and IL-6 in both groups. The Exp group exhibited significantly lower levels of both CA-125 (P<0.05) and IL-6 (P<0.05) compared to the Ctrl group.

**Table 1:** Serum levels of CA-125 and IL-6 in the Exp and Ctrl groups. Note: * suggested a significant difference with P < 0.05

Group	CA-125 (U/mL)	IL-6 (pg/mL)
Exp (LPS + DS)	28.5 ± 5.2	3.2 ± 1.1
Ctrl (LPS)	35.8 ± 6.8	4.8 ± 1.5
P-value	0.021	0.008

### Post-treatment QOL of patients

**Figure 4:**
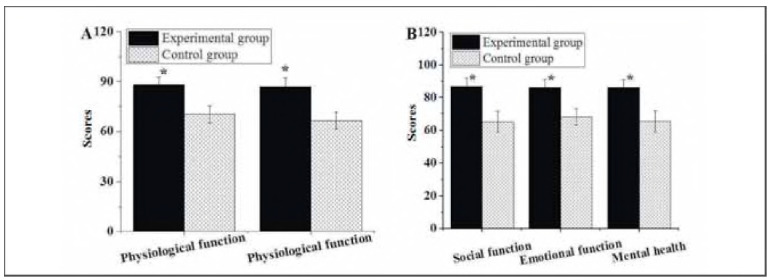
QOL assessment results of patients after various treatments.<br>(A: PF and PR scores; B: SF, EF, and EH scores)<br>Note: * suggested a great difference with P < 0.05

As summarised in Figure 4, the post-treatment PF score in the Exp group was 88.15±4.71, the PR score was 87.02±5.13 points, the SF score was 86.82±4.95 points, the Ef score was 85.96±5.33 points, and the EH score was 86.11±4.75 points. In the Ctrl group, the post-treatment PF, PR, SF, EF, and EH scores were 70.31 ±5.09 points, 66.48±5.11 points, 64.97±6.32 points, 68.01±4.97 points, and 65.22±6.42 points, respectively. It suggested that the post-treatment PF, PR, SF, EF, and EH scores in patients receiving LPS + DS were higher than those treated with LPS alone, exhibiting obvious differences (P<0.05).

### Social support of patients

**Figure 5:**
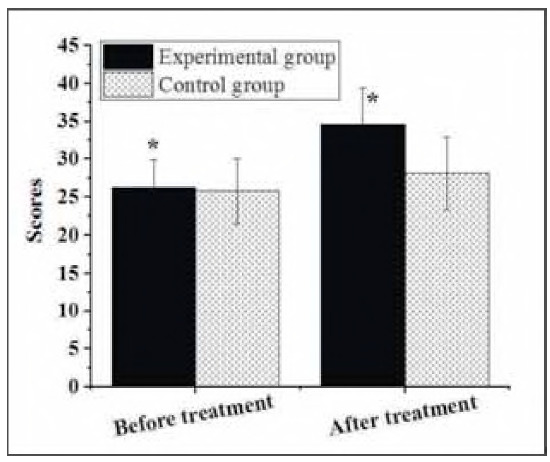
Pretreatment and post-treatment social support scores of patients.<br>Note: * suggested a great difference with P < 0.05

As demonstrated in Figure 5, the pretreatment and post-treatment social support scores in the Exp group were 26.18±3.67 points and 34.61 ±4.83 points, respectively, while, in the Ctrl group, those were 25.72±4.28 points and 28.06±4.77 points, respectively. No marked difference was observed in the pretreatment social support score between patients in the Exp and Ctrl groups (P>0.05). However, the post-treatment social support score of patients receiving LPS + DS was higher and showed a significant difference compared to that of patients treated with LPS alone (P<0.05).

### Family cohesion and adaptability of patients before and after they were treated differently

**Figure 6:**
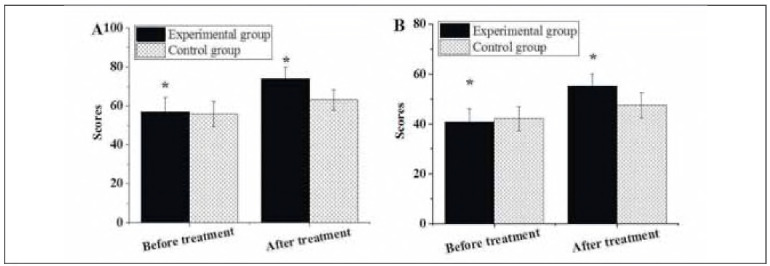
Family cohesion (A) and adaptability (B) of patients.<br>Note: * suggested a great difference with P < 0.05

The pretreatment family cohesion score in the Exp group was 57.13±7.06 points, and the family adaptability score was 40.85±5.35 points, while after treatment, they were 74.09±5.85 points and 55.11±4.93 points, respectively. In the Ctrl group, the pretreatment and post-treatment family cohesion scores were 55.88±6.43 points and 63.14±5.29 points, respectively, and the pretreatment and post-treatment family adaptability scores were 42.17± 4.85 points and 47.52±5.08 points, respectively. The above results were summarised and detailed in Figure 6. There was no obvious difference in pretreatment family cohesion and adaptability scores between patients in the Exp and Ctrl groups (P>0.05). Nevertheless, post-treatment family cohesion and adaptability scores in the Exp group were significantly higher than those in the Ctrl group, suggesting that patients treated with LPS + DS possessed closer family cohesion and adaptability (P<0.05).

### ARs of patients after they were treated differently

**Figure 7:**
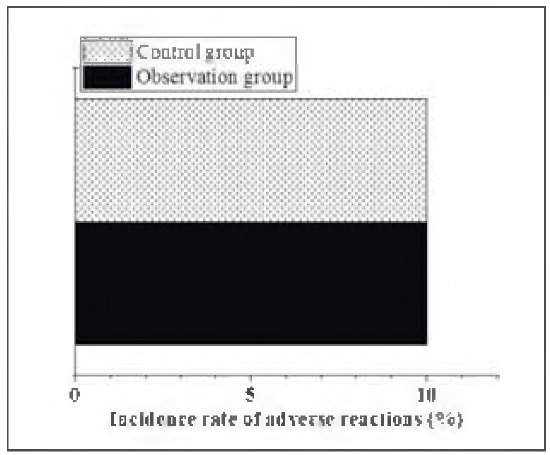
ARs of patients after they were treated differently.<br>Note: * suggested a great difference with P < 0.05

As illustrated in Figure 7, there was 1 case of vaginal bleeding, 3 cases of bruising, and 0 cases of subcutaneous hematoma in the Exp group. In the Ctrl group, there were 2 cases of vaginal bleeding, 2 cases of bruising, and 0 cases of subcutaneous hematoma. The incidence of ARs in the Exp group (10%) was not obviously different in contrast to that in the Ctrl group (10%) (P>0.05).

## Discussion

IVF-ET is a complex procedure in assisted fertility, preventing genetic disorders, and facilitating conception. Although IVF-ET is a crucial method for treating infertility, its success rate is still not ideal, and it can lead to various ARs [17] [18]. Therefore, data in this work were sourced from 80 patients who experienced repeated IVF failure and sought further assisted reproduction. Based on different treatment approaches, the patients were randomly assigned to two groups, with 40 patients in each. Patients in the Ctrl group with LPS treatment, while those in the Exp group treated with LPS + DS. Firstly, from a clinical data perspective, no significant differences were observed (P>0.05) in age, BMI, history of miscarriage, number of ETs, and educational level among patients in the different groups. This ensures the reliability of subsequent data analysis.

Pregnancy outcomes refer to the process starting from fertilisation of the egg and ending with the delivery of the fetus. This process includes miscarriage, induced abortion, fetal abnormalities, ectopic pregnancy, preterm birth, and other factors. Improving pregnancy outcomes is one of the key challenges that need to be further explored and addressed in assisted reproductive technology [19]. In this work, the Exp group had significantly higher CPR and EIR compared to the Ctrl group, while MCR was much lower than the Ctrl group, with marked differences (P<0.05). This suggests that the combination of LPS and DS can effectively improve pregnancy outcomes, increase CPR and EIR, and provide assistance to patients undergoing repeat assisted reproduction. Our findings demonstrated that the Exp group, treated with LPS + DS, exhibited significantly lower serum levels of both CA-125 and IL-6 compared to the Ctrl group. This is consistent with previous studies suggesting that DS can modulate these markers, potentially contributing to its beneficial effects on IVF outcomes. The reduction in CA-125 levels may indicate a decrease in inflammation and improved endometrial receptivity. In contrast, the lower IL-6 levels may suggest a suppression of the inflammatory response, creating a more favourable environment for implantation and maintaining pregnancy. Further research is needed to fully elucidate the mechanisms by which DS modulates these markers and their specific roles in IVF success.

In terms of hormone levels, the Exp group had lower FSH, E2, and LH levels compared to the Ctrl group, with statistically significant differences (P<0.05). The pituitary gland generates FSH levels, and high FSH levels may indicate reduced ovarian reserve or a lower number of remaining eggs. FSH levels can impact the success of fertility treatments [20]. Serum E2 measurement is a useful indicator for evaluating various menstrual abnormalities and monitoring induced ovulation and subsequent treatments in infertility patients. Elevated LH concentration is seen in conditions such as gonadal dysfunction, primary testicular failure, and inadequate tubular development, and low LH levels in both men and women can lead to infertility [21]. Therefore, the above results indicate that the combination of LPS and DS can effectively improve hormone levels in patients and help regulate the secretion of body hormones.

Although dalteparin does not directly target the hypothalamic-pituitary-gonadal axis, several mechanisms may explain its association with altered gonadotropin and estradiol levels. Low-molecular-weight heparin (LMWH) can enhance ovarian microcirculation by promoting nitric oxide (NO)-mediated vasodilation, potentially improving granulosa cell function and steroidogenesis [22]. Additionally, LMWH's anti-inflammatory and antithrombotic properties might reduce ovarian stromal inflammation and microthrombi, stabilising follicular development. Furthermore, by improving endometrial receptivity and early embryonic signalling, LMWH can influence pituitary feedback mechanisms, thereby fine-tuning gonadotropin release [23]. These hypotheses remain speculative, as direct studies on LMWH's impact on ovarian hemodynamics or pituitary feedback during the luteal phase of embryo transfer cycles are lacking. Future research, including Doppler ultrasound assessments of ovarian blood flow after LMWH administration and in vitro studies on granulosa cell responses, is necessary to elucidate whether the observed hormonal changes are direct pharmacological effects of dalteparin or secondary to improved early pregnancy physiology.

From the perspective of QOL, the Exp group exhibited remarkably higher scores in PF, PR, SF, EF, and EH, showing significant differences compared to the Ctrl group (P<0.05). This indicates that LPS, when combined with DS, can effectively improve patients' QOL. Social support refers to the objective support a person receives from their social relationships (family, friends, colleagues, etc.) and the subjective perception of that support [24]. In this work, the Exp group presented a higher social support score after treatment, with a statistically significant difference compared to the Ctrl group (P<0.05). This suggests that LPS, combined with DS, can effectively enhance a patient's ability to access social support. Furthermore, patients treated with LPS + DS showed higher scores in family cohesion and family adaptability compared to those treated with LPS alone, indicating statistically significant differences (P<0.05). This finding aligns with a study by Xing et al. (2020) [25], which indicates that LPS combined with DS improves family cohesion and adaptability, thereby enhancing the mental health of patients. The safety of DS is a significant concern in clinical practice. In this work, the incidence of ARs in the Exp group was 10%, which exhibited no great difference from that in the Ctrl group (10%) (P>0.05). This suggests that LPS and DS, when used jointly, do not increase the occurrence of ARs in patients and are considered safe and feasible. This study also examined the impact of LPS + DS on serum levels of CA-125 and IL-6, two markers that have been implicated in the pathogenesis of infertility and repeated implantation failure. The observed reduction in these markers suggests that LPS + DS may exert its beneficial effects, at least in part, by modulating the immune response and improving the endometrial environment.

Enoxaparin - a well-studied LMWH - has been tested in IVF patients, especially those with thrombophilia or recurrent implantation failure. In women with thrombophilia, a randomised trial showed that 40 mg/day of enoxaparin nearly quadrupled implantation rates (20.9% vs 6.1%), tripled clinical pregnancy rates (31% vs 9.6%), and increased live births (23.8% vs 2.8%) compared to placebo [26]. However, a large multicenter, double-blind trial in women with unexplained recurrent miscarriage found no improvement in live birth rates with enoxaparin versus placebo [27]. Together, these results suggest that the benefits of enoxaparin may be limited to specific high-risk subgroups rather than the broader IVF population. Dalteparin differs from enoxaparin chiefly in dosing convenience - its once-daily regimen can improve adherence compared to enoxaparin's twice-daily schedule - and in subtle institutional or clinician preferences. Both agents share similar safety profiles, so the choice often hinges on local availability, protocol traditions, and individual patient considerations.

## Conclusion

This study retrospectively collected data from 80 patients who underwent in vitro fertilisation (IVF) and embryo transfer (ET) at the hospital. These patients had experienced repeated IVF failure and required further assisted reproduction. They were randomly rolled into two groups, with 40 patients in the Exp and Ctrl groups, respectively, who were treated with LPS and LPS + DS, respectively. The findings revealed that LPS combined with DS effectively elevated hormone levels in patients and improved their quality of life, family cohesion, and adaptability. Furthermore, it did not increase the occurrence of ARs, demonstrating its safety and feasibility. However, this work had a small sample size of only 80 patients, and the data was obtained from a single source, which may have some impact on the results.

Additionally, the follow-up records for patient prognosis were not comprehensive enough, and the duration of follow-up was relatively short. Therefore, further case studies would be conducted to explore the therapeutic effects of DS in more depth. In addition to improved pregnancy outcomes and quality of life, this study demonstrated that LPS + DS can significantly reduce serum levels of CA-125 and IL-6, highlighting the potential immunomodulatory effects of this treatment. Consequently, this work yielded help for assisted reproduction treatments in patients with repeated IVF failures.

## Dodatak

### Conflict of interest statement

All the authors declare that they have no conflict of interest in this work.
